# Safety and immunogenicity of inactivated Rift Valley Fever Smithburn viral vaccine in sheep

**DOI:** 10.1186/s12985-023-02180-2

**Published:** 2023-10-03

**Authors:** Matome Selina Matsiela, Leeann Naicker, Thandeka Khoza, Nobalanda Mokoena

**Affiliations:** 1 Onderstepoort Biological Products (Pty. Ltd), 100 Old Soutpan Road, Onderstepoort, Pretoria, 0110 South Africa; 2 Department of Biochemistry, School of Life Sciences, University of KwaZulu-Natal (Pietermaritzburg Campus), Scottsville, 3209 KwaZulu-Natal South Africa

**Keywords:** Rift valley fever virus, Smithburn vaccine, Virus inactivation, Montanide™ Gel-01 adjuvant, Aluminium hydroxide adjuvant

## Abstract

**Background:**

The live-attenuated Rift Valley Fever Smithburn (SB) vaccine is one of the oldest products widely used in ruminants for control of RVF infections. Vaccinations with RVF Smithburn result in residual pathogenic effect and is limited for use in non-pregnant animals. Commercially available RVFV inactivated vaccines are considered safer options to control the disease. These products are prepared from virulent RVFV isolates and present occupational safety concerns. This research study evaluates the ability of an inactivated SB vaccine strain to elicit neutralising antibody response in sheep.

**Methods:**

The RVF Smithburn vaccine was inactivated with binary ethylenimine at 37 °C. Inactivated RVFV cultures were adjuvanted with Montande™ Gel-01 and aluminium hydroxide (Al (OH)_3_) gel for immunogenicity and safety determination in sheep. The commercial RVF inactivated vaccine and a placebo were included as positive and negative control groups, respectively.

**Results:**

Inactivated RVFV vaccine formulations were safe with all animals showing no clinical signs of RVFV infection and temperature reactions following prime-boost injections. The aluminium hydroxide formulated vaccine induced an immune response as early as 14 days post primary vaccination with neutralising antibody titre of 1:20 and a peak antibody titre of 1:83 was reached on day 56. A similar trend was observed in the animal group vaccinated with the commercial inactivated RVF vaccine obtaining the highest antibody titre of 1:128 on day 56. The neutralizing antibody levels remained within a threshold for the duration of the study. Merino sheep vaccinated with Montanide™ Gel-01-Smithburn were characterised with overall lower immune response when compared to aluminium hydroxide vaccine emulsions.

**Conclusions:**

These finding suggests that the inactivated RVF Smithburn vaccine strain adjuvanted with aluminium-hydroxide can be used an alternative to the products prepared from virulent RVFV isolates for protection of ruminants against the disease. The vaccine can further be evaluated for safety in pregnant ewes.

## Background

The Rift Valley Fever virus (RVFV) is a zoonotic arbovirus classified under the genus *Phlebovirus*, family *Phenuiviridae* and order Bunyavirales. It is a causative agent of an acute mosquito-borne viral disease that result in abortions in pregnant animals and neonatal mortalities [1–4]. The RVFV is considered a priority pathogen by the World Health Organization (WHO) and Centers for Disease Control and Prevention (CDC) due to its pathological effect in domestic ruminants and humans in African countries and neighbouring regions [5, 6]. Also, the RVFV is classified as Category A Priority Pathogen by the National Institute of Allergy and Infectious Diseases in the United States of America. If not effectively controlled, RVF poses a threat and has great potential to spread into non-endemic regions. Control and prevention of RVF outbreaks depends upon adequate management of sustainable animal vaccination programs [7].

Veterinary vaccines for protection of susceptible animals are commercially available (Table 1) and have been successfully used as effective means of controlling the spread of RVFV in endemic and high risk countries, such as, South Africa, the Arabian Peninsula, Kenya, Tanzania, Egypt and Sudan [8]. Vaccines against RVF are supplied as live-attenuated or inactivated products (Table 1). Live-attenuated vaccines are developed from virulent RVFV isolates following repeated passage in cultured cells to alter their virulence and ensure they do not cause disease in vaccinated animals [9]. These vaccines replicate in host cells and are able to induce long term protective immunity with a single dose [8]. This is consistent with efficacy reports of RVF Smithburn, Clone-13, MP-12, and thermostable Clone-13 vaccines which have been demonstrated to induce high immunity in ruminants [4, 8, 10]. However, safety concerns widely limit the application of live-attenuated vaccines, due to their potential of reversion to virulence.

**Table 1:** Commercially available RVF vaccines that are licensed for use in livestock

Vaccine	Type	Company	Route of administration/dose	Use in pregnant animals	References
Live Rift Valley Fever (Smithburn strain)	Live-attenuated	OBP, South Africa;	1 mL S/C in sheep, goat and cattle	No	[4, 20, 21]
RIFTVAX TM (Smithburn strain) vaccine	Live-attenuated	KEVEVAPI, Kenya)	1 mL S/C in sheep and goats, 2 mL in cattle	No	[4, 20, 21]
RVFV (Clone-13) vaccine	Live-attenuated	OBP, South Africa	1 mL S/C in sheep, goats and cattle	Yes	[4, 20, 21]
Formalin-inactivated RVFV (South African field strain)	Inactivated	OBP, South Africa	1 mL S/C in sheep and goats, 2 mL in cattle	Yes	[4, 20, 22]
Riftovax live-attenuated thermostable Clone 13 vaccine (CL-13T)	Live-attenuated	MCI Sante Animale, Morocco	1 mL S/C in sheep, goats and cattle	Yes	[21, 23]
BEI-inactivated RVFV (ZH501 strain) vaccine	Inactivated	VSVRI, Egypt	1 mL S/C in sheep and goats, 2 mL in cattle	Yes	[20, 21]

*OBP* onderstepoort biological products, *KEVEVAPI* Kenya Veterinary Vaccine Producing Institute, *VSVRI* Veterinary Serum and Vaccine Research Institute, *MCI* Multi-Chemical Industry, *BEI* binary ethylenimine, *S/C* subcutaneous, *ZH501* Zagazig Hospital RVFV strain 501

Live-attenuated vaccines have been reported to cause abortions and/or foetal malformations in pregnant ewes [8, 11–13]. The evidence of vertical transmission of the attenuated virus is in alignment with abortogenic and teratogenic properties of pathogenic strains [14]. Consequently, most live-attenuated RVF vaccines are limited for use in non-pregnant animals [11, 15, 16]. The inactivated vaccines are considered safer alternatives and may be used during pregnancy. These vaccines are produced by propagating live virus in cultured cells, followed by chemical, heat or radiation treatment to disable their ability to infect and replicate inside the host [17, 18]. The resultant product eliminates the aspect of possible reversion to virulence. Though inactivated RVF vaccines require booster dosing to induce protective immune responses, followed by repeated application on a yearly basis, they offer improved safety. Commercially available inactivated vaccines are manufactured from virulent field isolates. This poses occupational risk as the virus can be transmitted to humans through handling virulent RVFV material [19]. This study aims to improve the safety profile of the Smithburn live-attenuated RVFV vaccine strain while retaining its immunogenicity. The Smithburn virus vaccine was inactivated with BEI and formulated with Montanide™ Gel-01 and aluminium hydroxide adjuvants for evaluation of safety and immunogenicity in sheep.

## Methods

### Cells and virus

Adherent Baby Hamster Kidney (BHK-21) and African Green Monkey Kidney (Vero) cell lines, supplied by European Collection of Authenticated Cell Cultures (ECACC, UK) were maintained at 37 °C in a humidified incubator with 5% CO_2_. The cells were grown using standard cell culture techniques in Glasgow Minimum Essential Media (GMEM) (Gibco, USA) supplemented with 10% (v/v) bovine serum (Cell Sera, Australia) and antibiotics (100,000 U/L penicillin (Sigma Aldrich, USA), 100 mg/L streptomycin (Sigma Aldrich, USA) and 5,3 mg/ml amphotericin B (Sigma Aldrich, USA).

The RVF Smithburn virus strain used in the production of vaccine manufactured by Onderstepoort Biological Products SOC Ltd was obtained following internal procedures. The RVF vaccine strain was propagated in the BHK-21 cells in 850 cm^2^ roller bottles. The virus was quantified by viral plaque assays on Vero cell monolayers and results expressed as log_10_ PFU/ml [11].

### Production and inactivation of RVFV Smithburn

The live-attenuated RVFV Smithburn was produced in BHK-21 at 37 °C on a rolling apparatus and monitored daily until (2–3 days) 90% cytopathic effect has been reached. Infectious RVFV Smithburn was quantified by plaque forming unit (PFU) assay on Vero cell culture [24].

The binary ethyleneimine (BEI) was selected as an inactivating agent and prepared following the method described by Bahnemann [25]. The RVFV culture was used at a minimum titre of 1.00E + 06 PFU/mL and the following final concentrations of BEI were evaluated for inactivation of the Smithburn strain: 0.5 mM, 1 mM, 1.5 mM and 2 mM. Following the addition of each concentration of BEI to the RVFV Smithburn whole cell cultures, they were incubated at 37 °C with continuous stirring at 150 rpm for 24 h. Each concentration of BEI was prepared in more than one flask and evaluated at 2-h intervals. The untreated virus was included as the control. The BEI virus inactivation reaction was immediately neutralized by adding 1 M sodium thiosulphate pentahydrate (H_10_Na_2_O_8_S_2_) to the sampled aliquots at final concentration of 10% volume of BEI added. Neutralized inactivated samples were stored at 4 °C until tested for RVFV infectivity on cell culture. Inactivation was conducted in three independent experiments*.*

Virus inactivation was validated using the Real-Time Cell Analysis (RTCA) system (xCELLigence™, DP ACEA Biosciences Inc, US) as part of quality assurance [26]. The Vero cell suspension at 1–5 × 10^4^ cells/mL was seeded on the RTCA 16-well plates and a 100 µL of the culture media supplemented with 10% serum was added to make up a volume of 200 µL. Cell growth was monitored by recording the cell index (CI) hourly for up to 48 h to obtain exponential cell growth before infection with the inactivated-RVFV Smithburn aliquots. The 16-wellplates were incubated for 1 h at 37 °C with 5% CO_2_ following infection with 100 µL inactivated-RVFV aliquots. Cell culture media was added and the plates were further incubated at 37 °C with 5% CO_2_ and monitored on the RTCA system for up to 100 h. The Vero cell culture, untreated with the virus were included as the control in the analysis.

### Production of inactivated RVFV Smithburn vaccines

The RVFV Smithburn vaccine was prepared on BHK-21 cells and harvested virus culture was inactivated at the predetermined BEI concentration. Complete inactivation of the virus was confirmed and validated by infecting a monolayer of Vero cell culture with the killed Smithburn virus on 96-well microplates and 16-well RTCA E-plates. Two vaccine emulsions were formulated from the inactivated RVFV Smithburn cultures, one with aluminium hydroxide gel and the other with Montanide™ Gel 01 PR adjuvants (SEPPIC, France). The vaccine formulations were prepared under sterile conditions at room temperature using a high-speed homogenizer, D500 (Wiggens, China) at 13,000 rpm. Both vaccine formulations were subjected to standard quality control tests prior use.

### Animals, housing and care

Twenty merino sheep of mixed gender and between the ages of 6–12 months, were sourced from Langfontein farm in Mpumalanga, South Africa. The animals were pre-screened for presence of antibodies against RVFV using serological assays prior to the commencement of the trial. The serum neutralisation test (SNT) was conducted in-house at OBP and the samples were sent to Agricultural Research Council at the Onderstepoort Veterinary Institute for enzyme-linked immunosorbent assay (ELISA) [27, 28]. Animals that were free from RVFV antibodies were delivered to OBP for vaccine safety and immunogenicity studies. The sheep were housed in stables provided controlled environmental conditions at ambient temperature (20–25 °C), and lights switched off at night to mimic the natural environment. Animals were then allowed to acclimatize for 7 days prior the trial. Rectal temperatures of animals were recorded daily starting from the 4 days before beginning of trial. The animals were fed Epol Ram, Lamb and Ewe pellets, Eragrostis and allowed access to water *ad lib*. The stables were cleaned daily and wood shavings covering the floor replaced once a week. Animals enrolled in the study were identified by ear tags on the right-hand. Animal handling was done in accordance with the standard operating procedures at the experimental animal department at OBP.

### Safety and immunogenicity of inactivated RVFV Smithburn vaccines in sheep

Animals were assigned four groups (A, B, C and D), with each group containing 5 sheep for safety and immunogenicity studies. Groups A and B were allocated for the aluminium hydroxide and Montanide Gel-01 adjuvanted RVFV vaccinations, respectively. Individual animals were vaccinated via the subcutaneous (SC) route on the inner left thigh with 1 ml of the adjuvanted inactivated RVFV vaccines on day 0 and day 28 (Table 2). The animals in group C and D were for vaccination with the commercial inactivated RVFV vaccine supplied by OBP and sterile vaccine diluent, respectively, thus serving as positive and negative controls. The general clinical health of the individual animals, including the RVFV symptoms (such as fever, nasal discharge, loss of appetite, weakness, and bloody diarrhoea) were monitored and recorded once daily for the duration of the study. Rectal body temperatures of the animals were recorded at least once daily for 21 days post each vaccination.

**Table 2:** Vaccination of sheep with inactivated RVFV Smithburn formulation

Group	No. of animals	Vaccine	Dose of administration	Days of vaccination	Route of administration
A	5	Montanide™ Gel-01-RVFV (Smithburn)	1 ml	D0, D28	S/C
B	5	Aluminium hydroxide-RVFV (Smithburn)	1 ml	D0, D28	S/C
C	5	Commercial inactivated RVFV vaccine	1 ml	D0, D28	S/C
D	5	Sterile vaccine diluent	1 ml	D0, D28	S/C

### Blood collection

Each sheep participating in this study was bled in 4–5 mL clot activator and gel separating yellow top tubes on days 0, 7, 14, 21, 28, 35, 42, 49, 56 and 63 for serological analysis of humoral immune response. The blood collected was centrifuged at 2500× *g* at 4 °C for an hour. The serum samples were harvested into sterile cryovials and heated at 56 °C in a water bath for 30 min to inactivate non-specific viral inhibitory substances. The heat inactivated serum samples were stored at − 20 °C until used for serological analysis.

### Evaluation of the antibody immune response through Enzyme linked immunosorbent assay (ELISA)

The levels of IgM and IgG antibodies induced in sheep against the RVFV were estimated using ELISA assay, based on recombinant nucleoprotein (N protein). The ELISA was conducted at the ARC-OVI for concurrent detection of IgM and IgG antibodies against RVFV. The procedure was conducted according to the method described by Ellis et al. [27]. The net absorbance read at OD_(450 nm)_ was expressed as S/P% of the positive control, and all serum samples with S/P% of ≥ 7 were considered positive [27].

### Evaluation of the antibody immune response through serum neutralisation test (SNT)

The SNT assay was used to detect the presence of specific neutralising antibodies in serum of vaccinated sheep. The antibody levels measured by this test are mostly induced against the Gn/Gc glycoproteins of RVFV and afford protection in infected and vaccinated animals. The SNT method was performed as described by Frey and Liess [28]. Briefly, the heat inactivated serum samples were serially diluted two-fold using GMEM cell culture media supplemented with streptomycin and amphotericin B antibiotics (Sigma Aldrich, USA). Positive and negative anti-RVFV serums, were included as controls. The live RVFV Smithburn was added in each well (50 µL) to achieve a final concentration of 100 TCID_50_/ml. After 1-h incubation of the sera with the virus at 37 °C, 100 µL of Vero cells were added in 96-well microtitre plates. The cell monolayer was examined for presence of CPE under light microscope after four days. Antibody titres were expressed as the reciprocal of the serum dilution that inhibited 50% of virus-induced CPE [29].

### Ethical approval

The veterinary clinical trial experiment was performed according to the approved protocol by the OBP Animal Ethics Committee (South African Veterinary Council Facility Registration Number: FR1514054) under approval number OBP2019/002 and Department of Agriculture, Land Reform and Rural Development under section 20 of Animal Diseases Act (Act 35 of 1984); approval number 12/11/1/1/(b) MG.

### Statistical analysis

Statistical analysis was conducted to compare the significant difference of the antibody immune response induced in sheep by the inactivated RVFV Smithburn formulations. A two-way analysis of variance (ANOVA) followed by a post hoc Tukey’s test was utilized to determine difference in antibody immune response over time. Graphpad prism version 5.0 software for Windows was utilized for statistical analysis at a significance level of 5% (*p* < 0.05).

## Results

### Inactivation profile of RVFV using binary ethylenimine

**Fig. 1:**
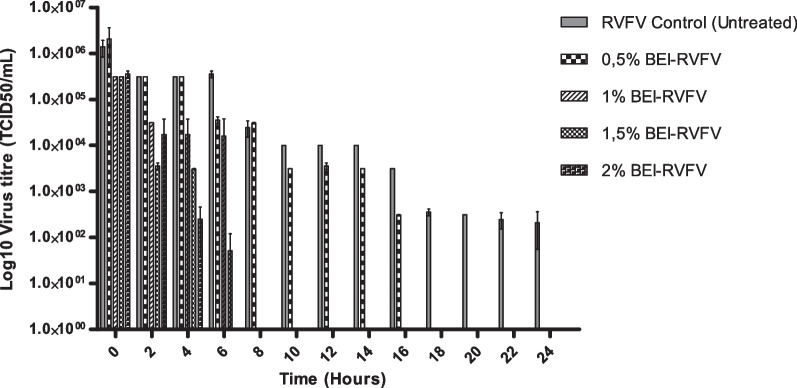
Inactivation profile of Rift valley fever virus (RVFV) with binary ethylenimine (BEI). Average titres of the 2-h interval RVFV (Smithburn) inactivated with 4 different concentrations of BEI (0.5 mM, 1 mM, 1.5 mM and 2 mM) were determined. Titres were measured using the tissue culture infectious dose (TCID50) assay. RVFV culture was included and used as a negative control. Complete inactivation of RVFV was first observed at BEI concentration 2 mM after 6 h of treatment, followed by 1.5 mM and 1 mM BEI after 8 h of treatment. Complete inactivation of RVFV by 0.5% BEI was observed after 18 h of treatment. Error bars represent standard error of the mean (SEM)

**Fig. 2:**
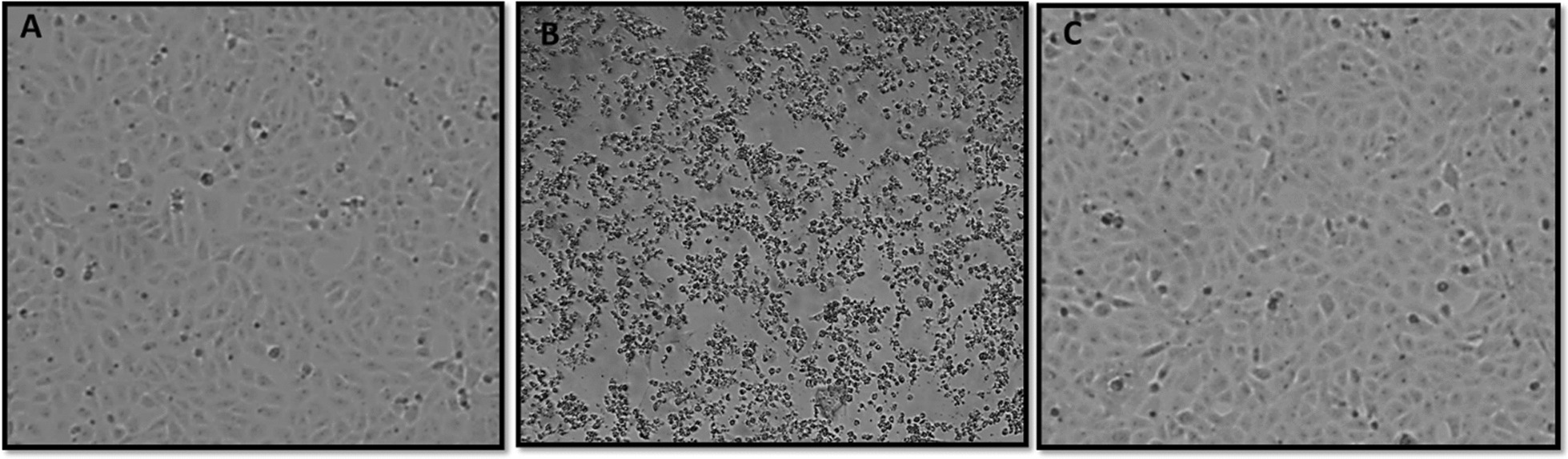
Monolayer of Vero cell culture on the fifth day of incubation after treatment with Rift Valley Fever virus (RVFV) Smithburn. **a** Non-treated cell control, **b** cells treated with the lowest dilution of the live RVFV Smithburn, **c** cells treated with the lowest dilution of the inactivated RVFV. Cytopathic effect was not observed in Vero cells infected with the lowest dilution of the inactivated RVFV. The non-treated cell culture and live RVFV were respectively used as positive and negative controls

The inactivation of RVFV Smithburn was evaluated at different concentrations of BEI (0.5 mM, 1 mM, 1.5 mM and 2 mM). The absence of virus infectivity was determined as a function of TCID_50_ titres, for selection of a suitable BEI concentration that completely inactivates RVFV. In addition, the virus inactivation was validated by measuring the inability of inactivated RVFV culture to induce cell death following incubation at 37 °C. Inactivation kinetics of RVFV after treatment with different concentrations of BEI are demonstrated on Fig. 1. The data showed that higher concentrations of BEI required a short time period to achieve complete inactivation when compared to lower concentration. The lowest BEI concentration (0.5 mM) completely inactivated RVFV Smithburn following 18-h incubation period as indicated by no CPE on Vero cells after incubation. All other BEI concentrations higher than 0.5 mM (1 mM, 1.5 mM and 2 mM) achieved complete inactivation after 8 h of treatment, with higher concentration 2 mM achieving inactivation after 6 h of treatment (Fig. 1). This data was supported by absence of CPE after infecting monolayers of Vero cells with the lowest log dilution of the inactivated virus culture (cell morphology presented on Fig. 2). The untreated RVFV control induced continuous CPE on infected cells from 0 up to 24 h post incubation, and reduced virus titre with time as a result of incubation at 37 °C. The overall results indicated that the rate of RVFV inactivation is directly proportional to the concentration of BEI used in the inactivation process.

### Preparation of the inactivated RVFV Smithburn vaccine pilot batch

**Fig. 3:**
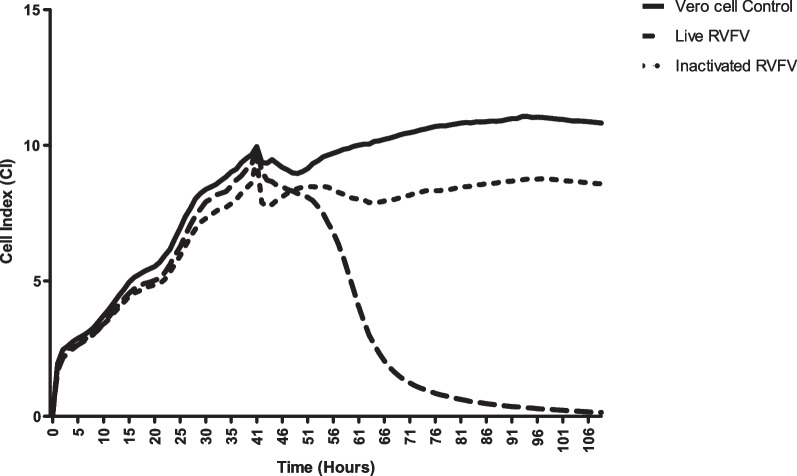
Validation of the inactivation of rift valley fever virus (RVFV) Smithburn using real time cell analysis (RTCA) assay. Growth profile of Vero cells infected with the inactivated and live RVFV Smithburn was recorded. No cell death was recorded in Vero cell culture infected with the inactivated RVFV Smithburn as indicated by the stationary cell index following treatment. The live RVFV induced a decline in cell index showing cell death following treatment with the virus. Vero cell culture was included as positive control

A pilot batch of RVFV Smithburn was prepared and inactivated with 0.5 mM BEI at 37 °C for 24 h. Inactivated vaccine used to infect BHK-21 monolayer resulted in no CPE following 5 days of incubation at 37 °C (Fig. 2). A monolayer of cells non-treated with the virus and cells treated with live RVFV were included as controls. The live RVFV induced CPE in infected Vero cell culture and no CPE was observed with cells only control. The cells infected with the inactivated RVFV Smithburn vaccine were comparable with the cells only control (Fig. 2). Complete inactivation of the virus was further validated using the RTCA by monitoring the cell index (CI) of Vero cell culture to indicate various growth phases before and after treatment with inactivated RVFV [18]. The CI profile of Vero cells seeded on E-plate wells followed a typical growth curve with CI value of 0–10 in the first 48 h of the experiment. The CI reached as high as 10 indicating absence of virus infectivity in cells following 48-h incubation on the RTCA system (Fig. 3). Following infection of cells with live RVFV, a gradual decrease in the CI value was observed from 50 to 130 h of incubation due to CPE formation of infected cells (Fig. 3). The CI of cells treated with inactivated RVFV followed a typical growth phase as the cell only control indicating complete inactivation of the RVFV Smithburn (Fig. 3). The slight difference in CI values between cells infected with inactivated virus and cells only control was attributed to the media components added. The inactivated virus contained neutralized BEI reaction mix whereas the cells only controls were topped up with fresh GMEM media which provided more nutrients.

The RVFV culture was split into two aliquots and formulated with aluminium hydroxide gel and Montanide™ Gel-01 adjuvants to achieve two vaccine emulsions. The vaccines were formulated at the same dose as the commercial inactivated RVF vaccine. Inactivated vaccine was tested for sterility before and after formulation using the standard test methods. Vaccine emulsions were tested on blood tryptose agar with bovine blood, and inoculated on thioglycolate and soy media. Sterility test revealed that the vaccines were free from bacterial and fungal contamination as confirmed during the period of observation after incubation on designated media for 14 days.

### Safety evaluation of the inactivated RVFV Smithburn vaccine in sheep

**Fig. 4:**
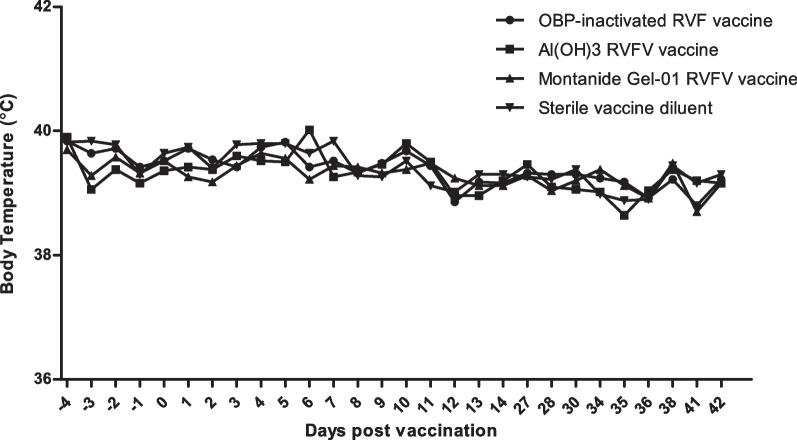
Average body temperatures of sheep vaccinated with the inactivated Rift valley Fever virus (RVFV) vaccines. Sheep retained the average normal temperature range of 38.3–39.9 °C, suggesting all inactivated vaccine formulations are safe for use in target species

Merino sheep were vaccinated with inactivated RVFV Smithburn vaccines. The commercial inactivated RVFV vaccine and sterile vaccine diluent served as positive and negative controls, respectively. Merino sheep were monitored daily for clinical signs of infection, reduced mobility and possible mortalities. Rectal temperatures were recorded 4 days prior the start of the trial and 14 days post each vaccination. Animals in all groups showed no signs of distress nor temperature increments following vaccination. The mean temperature records of the four groups of animals are indicated in Fig. 4. All the vaccinated and placebo animals maintained a normal range of the physiological temperature of sheep (38–40 °C) following primary and secondary vaccination with the inactivated RVFV Smithburn vaccine (Fig. 4). No detectable clinical signs of RVFV infection, or other signs of localized inflammations on vaccination sites, nor mortalities recorded in vaccinated animals.

### Immunogenicity of the inactivated RVFV vaccine in sheep

To evaluate the immunogenicity of the inactivated RVFV vaccines in sheep, antibodies against RVFV were measured in serum collected at 7-days interval from 0 to 63 days post vaccination. The ELISA method was primarily applied to estimate levels of IgM and IgG antibodies. The ≥ 7 S/P% is indicative of positive immune response for IgM and IgG antibodies [27]. The neutralizing antibody titres of ≥ 1:4 when using SNT method are indicative of seroconversion and ≥ 1:16 is considered sufficient to confer protection against the RVF disease as per OBP internal standard operating procedures.

#### Determination of the IgM antibody level

**Fig. 5:**
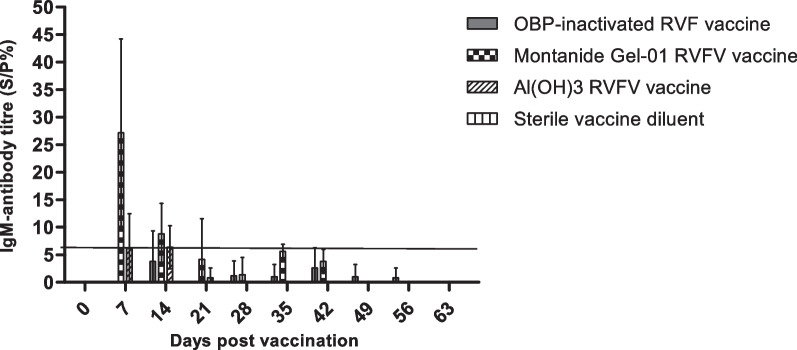
Total IgM antibody levels conferred by the inactivated Rift Valley Fever virus (RVFV) vaccines in sheep. IgM antibody titres against the RVFV nucleoprotein were measured using ELISA. Serum samples were collected from sheep injected with inactivated RVFV vaccine candidates at 0, 7, 14, 21, 28, 35, 42, 49, 56 and 63 days post vaccination. The commercial OBP-inactivated RVF vaccine and sterile vaccine diluent were respectively included as positive and negative controls. Data sets are presented as the mean ± SEM in the same treatment

Two groups of animals were used to determine and compare the immunogenicity of the inactivated RVFV Smithburn vaccine formulations, namely a Montanide™ Gel 01-SB and Al(OH)_3_-SB. The commercial inactivated RVFV vaccine and the sterile vaccine diluent were included as positive and negative controls, respectively. The results presented in Fig. 5 showed that the IgM antibodies against RVFV were not induced in the animal group vaccinated with placebo vaccine (sterile vaccine diluent). A low IgM antibody immune response, below the threshold was observed in animals vaccinated with the commercial inactivated RVFV vaccine on days 14–49 post vaccination (Fig. 5). The Al(OH)_3_-SB vaccine induced marginal IgM antibody immune response on day 7 and 14 days of primary vaccination. The highest neutralising IgM antibody levels of 27 S/P% was observed in animals vaccinated with Montanide™ Gel-01-SB vaccine emulsion after 7 days of primary vaccination (Fig. 5). The antibody levels gradually decreased to 1 S/P% after 28 days of primary vaccination.

#### Determination of the IgG antibody level

**Fig. 6:**
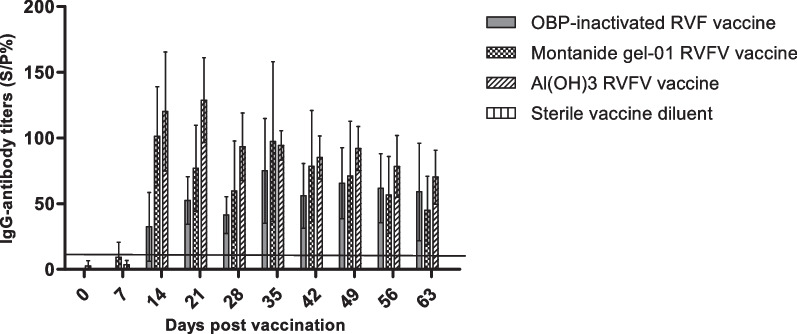
Total IgG antibody levels conferred by the Rift Valley Fever virus (RVFV) formulations in sheep. IgG antibody titres against the RVFV nucleoprotein were measured using ELISA. Serum samples were collected from sheep injected with inactivated RVFV vaccine candidates at 0, 7, 14, 21, 28, 35, 42, 49, 56 and 63 days post vaccination. The commercial OBP-inactivated RVF vaccine and sterile vaccine diluent were respectively included as positive and negative controls. Data sets are presented as the mean ± SEM in the same treatment

Figure 6 represents data obtained for IgG antibody immune response induced in sheep immunized with the inactivated RVFV Smithburn vaccine candidates. Sheep injected with Al(OH)_3_-SB vaccine obtained higher IgG antibody immune response (120 S/P%) at day 14 post primary vaccination (Fig. 6). The IgG antibodies in this group of animals were maintained throughout the trial with a peak antibody response (128 S/P%) observed at day 21 post primary vaccination (Fig. 6). The animal group immunized with Montanide™ Gel-01-SB vaccine induced IgG-antibody immune response (101 S/P%) after 14 days of primary vaccination. A 32 S/P% IgG antibody response was detected in the positive control animal group immunized with the commercial inactivated vaccine after 14 days of primary vaccination, with a peak antibody response (75 S/P%) observed after 7 days of booster vaccination (Fig. 6). No IgG antibodies were detected in the animal group vaccinated with the placebo vaccine throughout the trial (Fig. 6). Both inactivated vaccine formulations induced IgG response that was comparable to the commercial vaccine and remained above the threshold throughout the duration of the trial.

### RVFV-neutralising antibody titre determined by SNT

**Fig. 7:**
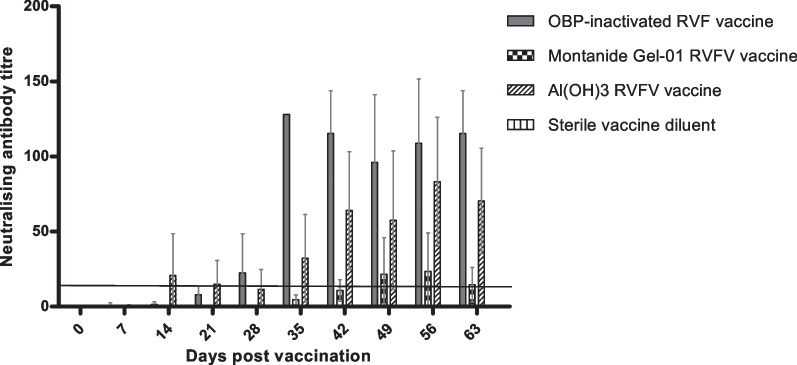
Neutralizing antibody immune response conferred by the inactivated RVFV vaccines in sheep. Neutralizing antibody titres were measured using serum neutralization test for day 0, 7, 14, 21, 28, 35, 42, 49, 56 and 63 post vaccination. The commercial OBP-inactivated RVF vaccine and sterile vaccine diluent were respectively included as positive and negative controls. Error bars represent standard error of the mean (SEM)

SNT tests were conducted to determine neutralising antibody titres against-RVFV in sheep and results were expressed as reciprocal of the dilution which neutralized 50% of the RVFV. Sheep injected with the commercial inactivated RVFV vaccine (manufactured by OBP) developed neutralising antibody titres of 1:22 after 28 days of primary vaccinations. The vaccine reached the peak antibody titre (1:128) 7 days following booster vaccination, and the antibodies were maintained up to the end of the trial (day 63) (Fig. 7). No detectable neutralising antibody response was observed for the negative animal control group vaccinated with sterile vaccine diluent. The Montanide™ Gel-01-SB vaccine emulsion on the other hand induced low neutralising antibody titres in sheep with a peak antibody response (1:23) on day 56 post primary vaccination (Fig. 7). The Al(OH)_3_-SB vaccine emulsion seroconverted with 1:21 from day 14 post primary vaccination with peak neutralising antibody response (1:83) observed on day 56 post vaccination (Fig. 7). The antibody titres induced by this vaccine in sheep were maintained throughout the duration of the trial (Fig. 7).

## Discussions

RVF is an important zoonotic viral disease that affects livestock and humans. Effective prevention and control of the spread of RVFV in endemic countries is achieved through vaccination of susceptible animals [30]. Livestock vaccination programs result in reduced outbreaks and transmission to humans. Live-attenuated RVFV Smithburn vaccine is one of the commercially available vaccines recommended for use to prevent RVF outbreaks. However, this vaccine strain is associated with several limitations such as possible reversion to virulent state of the virus, teratogenicities as well as abortions in pregnant animals [11]. Inactivated vaccines have been demonstrated to be safe for use in pregnant animals but are manufactured from virulent field isolates presenting the risk of infection for individuals processing the vaccine strains prior to inactivation. The vaccine drawbacks present a need for research towards the development of suitable RVFV vaccine candidates with improved safety and immunogenicity to protect against the spread of the virus in livestock, and safe for handling by laboratory personnel.

Inactivated vaccine improvement is dependent on the type of inactivating agent and the adjuvant used for vaccine formulation [31]. This study evaluated the immune response of sheep vaccinated with the BEI-inactivated RVFV Smithburn vaccine. BEI is an inactivating agent previously introduced for inactivation of viruses in vaccine production, and it was shown to be more advantageous for use as an alternative to formalin due to its protein preservative effect [32]. Inactivation of RVFV Smithburn using BEI was evaluated at 0.5 mM, 1 mM, 1.5 mM and 2 mM, and complete inactivation was observed in all BEI concentrations in test within 24 h of treatment. BEI was therefore used at the lowest concentration of 0.5 mM for 24 h of treatment to ensure complete inactivation of RVFV while preserving viral proteins. Two vaccine formulations of the RVFV were prepared using Montanide™ Gel 01 and aluminium hydroxide gel adjuvants, to select a suitable adjuvant for formulation of the inactivated RVFV-SB vaccine. The vaccines were prepared at the same dosage as the commercial inactivated vaccine derived from RVF HB74 virulent strain manufactured by OBP. Both inactivated RVFV Smithburn formulations were subjected to the standard vaccine quality control process to ensure that they met specifications for freedom from extraneous agents, safety and potency, prior to use in clinical trials. Safety and immunogenicity of the inactivated RVFV Smithburn emulsions were evaluated in target Merino sheep, following primary and secondary vaccination. No local reactions and clinical side effects were observed after each inoculation of vaccine in animals. Safety of the commercial inactivated RVF has been established extensively [17], and in this study as anticipated the vaccine induced no reaction with all 5 sheep injected. No febrile reactions were observed nor any form of discomfort in animals. Body temperatures of the animals were within a normal range of the physiological temperature after each inoculation. A study conducted by Magd et al. [33] also demonstrated safety of the BEI inactivated RVFV vaccine formulated with 20% Montanide Gel-01 and aluminium hydroxide, which resulted in no mortalities nor clinical signs of RVFV infections in mice. Safety test results suggests that the inactivated RVFV formulated with aluminium hydroxide gel and Montanide™ Gel-01 are safe for use in sheep following primary and secondary vaccinations.

The humoral immunogenicity of the vaccine formulations, in the form of IgM and IgG were monitored and analysed at 7-day intervals from day 0 up to day 63 post vaccination. The neutralising IgM antibodies were detected from 7 to 14 days post primary vaccination in animal groups vaccinated with Montanide™ Gel-01-SB vaccine and Al(OH)3-SB vaccine emulsions. The animal group vaccinated with Montanide™ Gel-01-SB vaccine obtained high positive IgM antibody levels, which were shown to decrease to negative response from day 21 after primary vaccination until the end of the trial. This was expected as the Montanide gel adjuvant is known to induce strong short term inflammatory immune response to trigger an efficacious response, and this was evident in our study as highest levels of IgM were obtained with this adjuvant. Several studies have also described IgM antibodies as a form of early onset of immunity which appear briefly as early as 3 days in response to infection and decreases few days following accumulation of IgG antibodies in circulation [34, 35]. Wallace et al. [35], has reported detection of IgM antibodies following 3 days post challenge with virulent RVFV M35/74 strain, which were shown to rapidly decrease from day 9–14 due to an increase in IgG antibody levels (14–21 days post challenge). Similar observations occurred in our study where the IgM antibodies were detected 7–14 days post primary vaccination of sheep with Montanide™ Gel-01-SB vaccine and Al(OH)_3_-SB vaccine emulsions. These antibodies decreased to negative levels following accumulation of IgG antibodies depicted from 14 days post primary vaccination (Figs. 5, 6) which is a typical profile of adaptive immune response. The animal group vaccinated with Montanide™ Gel-01-SB vaccine induced IgG antibody levels after 7 days post primary vaccination as determined with an ELISA method. The antibodies were maintained at high levels from day 14 until end of the trial (Fig. 6). Literature has reported that IgG antibodies begin to accumulate in circulation after 7 days of infection or vaccination, and this was in accord with data obtained after vaccination of sheep with Montanide™ Gel-01-SB vaccine. The animal group vaccinated with Al(OH)_3_-SB vaccine emulsion induced highest IgG antibody immune response when compared to both Montanide™ Gel-01-SB vaccine emulsion and the commercial inactivated RVFV (Fig. 6). The antibody immune response induced by Al(OH)_3_-SB vaccine remained at high levels from day 14 until end of the trial. The commercial inactivated RVFV vaccine had induced low IgM antibody titres in sheep 14 days post primary vaccination, which were below the threshold of 7 S/P%. However, the vaccine induced positive IgG antibodies which were detected 14 days post primary vaccination, with peaks observed following 7 days post booster vaccination and remaining at high levels until the end of the study. Similar results were obtained in a study reported by Ronchi et al. [36] who had detected negative IgM antibodies and high IgG antibodies against RVFV 7 days after vaccination of sheep with an inactivated RVFV vaccine formulated with 10% Montanide Pet Gel A. The vaccine reported by Ronchi et al. [36], and OBP commercial inactivated product are both formulated from virulent RVFV strains.

The high levels of IgG antibodies are an indication of a long-term immune response induced in sheep following vaccination. These IgG antibodies are traditionally considered to correlate with protective humoral immune response to viruses, when characterized with neutralising capabilities [37]. The ELISA data generated in this study measured antibodies induced against the N protein of RVFV which is known to be the most immunogenic protein within the *Phenuiviridae* family. Several studies have also reported that the epitopes exposed on the N protein are not involved in mediating virus attachment and entry, and thus may not induce neutralising antibody immune response [38–40]. Additional assays were employed to make an informed decision on potential efficacy of inactivated RVF Smithburn vaccine candidates. Therefore, serum neutralising test against RVFV was included as confirmatory assay to detect neutralising antibodies induced by the RVF SB vaccine candidates. The commercial inactivated RVFV vaccine conferred the highest neutralising antibody immune response observed 28 days after primary vaccination (Fig. 7). The Al(OH)_3_-SB vaccine emulsion conferred neutralising antibody immune response as early as 14 days after primary vaccination and the response increased following booster dose. The antibody titres were maintained at protective levels until the end of the trial. There was no significant difference in antibody titres induced by commercial inactivated RVFV vaccine and Al(OH)_3_-SB vaccine with *P* = 0.0004 and *P* = 0.0003 for ELISA and SNT data, respectively. This data suggests the inactivated Al(OH)_3_-SB formulation might result in similar protection levels to the current OBP inactivated RVF commercial vaccine product since they were evaluated at the same dosage and formulated with same adjuvant. The slight difference in antibody levels obtained with SNT and IgG ELISA for both Al(OH)_3_-SB emulsion and the commercial inactivated RVFV vaccines can be associated with the use of different RVFV strains used in the formulation. The commercial inactivated RVFV vaccine induced highest neutralising antibodies through SNT which may be explained by fact that the vaccine is prepared from formulation with virulent RVFV when compared to the attenuated SB vaccine strain. The IgG levels induced by Montanide™ Gel-01-SB vaccine comprised of low levels of neutralising antibodies as depicted in Fig. 7 when compared to aluminium hydroxide gel vaccine products. The Montanide™ Gel-01-SB vaccine emulsion induced high level of non-neutralising antibodies specific to RVFV and requires further evaluation in virulent virus challenge studies. The results generated in this study proved the concept that inactivated RVF vaccine prepared from attenuated Smithburn strain adjuvanted with aluminium hydroxide gel can potentially serve as an alternative candidate vaccine for protection of livestock against RVF.

## Conclusions

Immunogenicity and safety of an inactivated RVFV Smithburn was evaluated in sheep after primary and secondary inoculations. The results obtained indicated that the inactivated RVFV Smithburn formulated with aluminium hydroxide gel conferred strong neutralising antibody immune response in sheep. Though challenge with virulent RVFV strain was not conducted, there was no significant statistical difference in antibody titres between commercial inactivated RVFV vaccine and Al(OH)_3_-SB formulated product, suggesting the potential of this formulation to confer protection similar to the current OBP inactivated RVF vaccine. This vaccine formulation may be further developed and evaluated into a full vaccine product that confer protection against RVFV challenge, including duration of immunity studies. The safety of the vaccine may further be evaluated in pregnant animals.

## Data Availability

The datasets and relevant reagents used during the current study are available from the corresponding author following the legal process of the Onderstepoort Biological Products policies on material distribution.
